# Laserspritzer: A Simple Method for Optogenetic Investigation with Subcellular Resolutions

**DOI:** 10.1371/journal.pone.0101600

**Published:** 2014-07-03

**Authors:** Qian-Quan Sun, Xinjun Wang, Weiguo Yang

**Affiliations:** 1 Department of Zoology and Physiology, University of Wyoming, Laramie, Wyoming, United States of America; 2 Graduate Neuroscience Program, University of Wyoming, Laramie, Wyoming, United States of America; CSIC-Univ Miguel Hernandez, Spain

## Abstract

To build a detailed circuit diagram in the brain, one needs to measure functional synaptic connections between specific types of neurons. A high-resolution circuit diagram should provide detailed information at subcellular levels such as soma, distal and basal dendrites. However, a limitation lies in the difficulty of studying long-range connections between brain areas separated by millimeters. Brain slice preparations have been widely used to help understand circuit wiring within specific brain regions. The challenge exists because long-range connections are likely to be cut in a brain slice. The optogenetic approach overcomes these limitations, as channelrhodopsin 2 (ChR2) is efficiently transported to axon terminals that can be stimulated in brain slices. Here, we developed a novel fiber optic based simple method of optogenetic stimulation: the laserspritzer approach. This method facilitates the study of both long-range and local circuits within brain slice preparations. This is a convenient and low cost approach that can be easily integrated with a slice electrophysiology setup, and repeatedly used upon initial validation. Our data with direct ChR2 mediated-current recordings demonstrates that the spatial resolution of the laserspritzer is correlated with the size of the laserspritzer, and the resolution lies within the 30 µm range for the 5 micrometer laserspritzer. Using olfactory cortical slices, we demonstrated that the laserspritzer approach can be applied to selectively activate monosynaptic perisomatic GABAergic basket synapses, or long-range intracortical glutamatergic inputs formed on different subcellular domains within the same cell (e.g. distal and proximal dendrites). We discuss significant advantages of the laserspritzer approach over the widely used collimated LED whole-field illumination method in brain slice electrophysiological research.

## Introduction

Optogenetic approaches have become the method of choice to manipulate neuronal excitability *in vitro* and *in vivo*. The modular design of molecular optogenetic components enables bidirectional and cell-specific control of neuronal excitabilities [1]–[5]. This high degree of molecular specificity spawns the unprecedented ability to manipulate brain activities. Rapid progress has been made to improve the molecular design of optogenetic tools to make the process more suitable to the various needs of interrogation. For example, advances in molecular engineering have created optogenetic reagents with improved channel biophysical properties, optical properties, cell-specific expression, and subcellular delivery *in vivo* [1], [4], [6]–[9]. Meanwhile, major technological advancements have transpired to achieve better spatial resolution in light delivery [10]–[13], simultaneous optical stimulation, and electrical recordings *in vivo* [9], [11], [14], [15] and *in vitro* [15].

Brain slice preparations have been used extensively to help understand circuit wiring within specific brain regions. To build a detailed circuit diagram, one needs to measure functional synaptic connections between specific types of neurons. A high-resolution circuit diagram should provide detailed information at the subcellular levels (e.g. distal vs. basal dendrites). During the past few decades, electrophysiological recordings from connected cortical neurons in brain slices have demonstrated many intra- and inter- laminar connections between specific neuronal subtypes [16]–[20]. However, a limitation lies in the difficulty of studying long-range connections between brain areas separated by millimeters, because long-range connections are likely to be cut in a 300µm brain slice [21], [22]. The optogenetic approach overcomes these limitations as ChR2 is efficiently transported to axonal terminals that can be reliably stimulated in brain slices, even when axons are cut. Approximately 90% of interneuron dendrites and 70% of pyramidal neuronal dendrites are confined within the 150 µm range, which can be maintained in a 300 µm slice preparation. If the dendrites of the recorded cell remain largely intact in a brain slice, the strength of long-range inputs can be unequivocally induced by blue light stimulation and recorded. Combined with pharmacological approaches to block action potential propagation (TTX and 4-AP), activation of monosynaptic inputs can be achieved [13], [23].

For brain slice neural circuit interrogations, ideal optogenetic tools should have the following features [24]: *1)* subcellular resolution such that synaptic currents formed on specific cellular compartments can be selectively interrogated [e.g. sCRACM method, [13], [25], [26]] or the two photon activation approach [10], [27], [28]; *2)* a high degree of cell or synaptic specificity; and 3) ease of use with existing electrophysiology setups. So far, the two photon ChR2 activation approach retains the best spatial resolutions (<10 µm). However, this approach requires genetically modified opsin tools and an expensive two photon setup [28]. The subcellular channel-rhodopsin-assisted circuit mapping (sCRACM) approach is a cutting edge tool designed specifically for circuit mapping within brain slice preparations. In this approach, photo-activation of ChR2 is performed by shuttering (1.0 ms pulse) the beam of a blue laser in the specimen plane via a 5x objective. The movement of the laser beam is precisely controlled with mirror galvanometers (Cambridge Technology), triggered by scanning and data acquisition software Ephus (http://www.ephus.org)[29]. The sCRACM approach possesses adequate spatial resolutions (around 50 µm), the ability to scan a large area (millimeters), and the ability to activate all synaptic inputs formed onto different subcellular compartments of the recorded neuron [13]. However, this approach requires a setup of specialized optical equipment which requires substantial modification of the recording setup, and in depth knowledge of optics and the Matlab based program Ephus [29]. These limitations hinder the application of these cutting edge technologies.

To combat these limitations, a simple and low-cost novel approach is described below. This is a straight forward fiber optic based local light delivery method that we named laserspritzer; analogous to the picospritzer used by many electrophysiology labs for locally delivering drugs within a small area. We provide experimental data to demonstrate its spatial properties and its application in circuit investigations using olfactory cortical slices. We also discuss the advantages of the laserspritzer over the widely used collimated LED whole-field illumination method.

## Materials and Methods

### The laserspritzer approach: methods and validations

**Figure 1:**
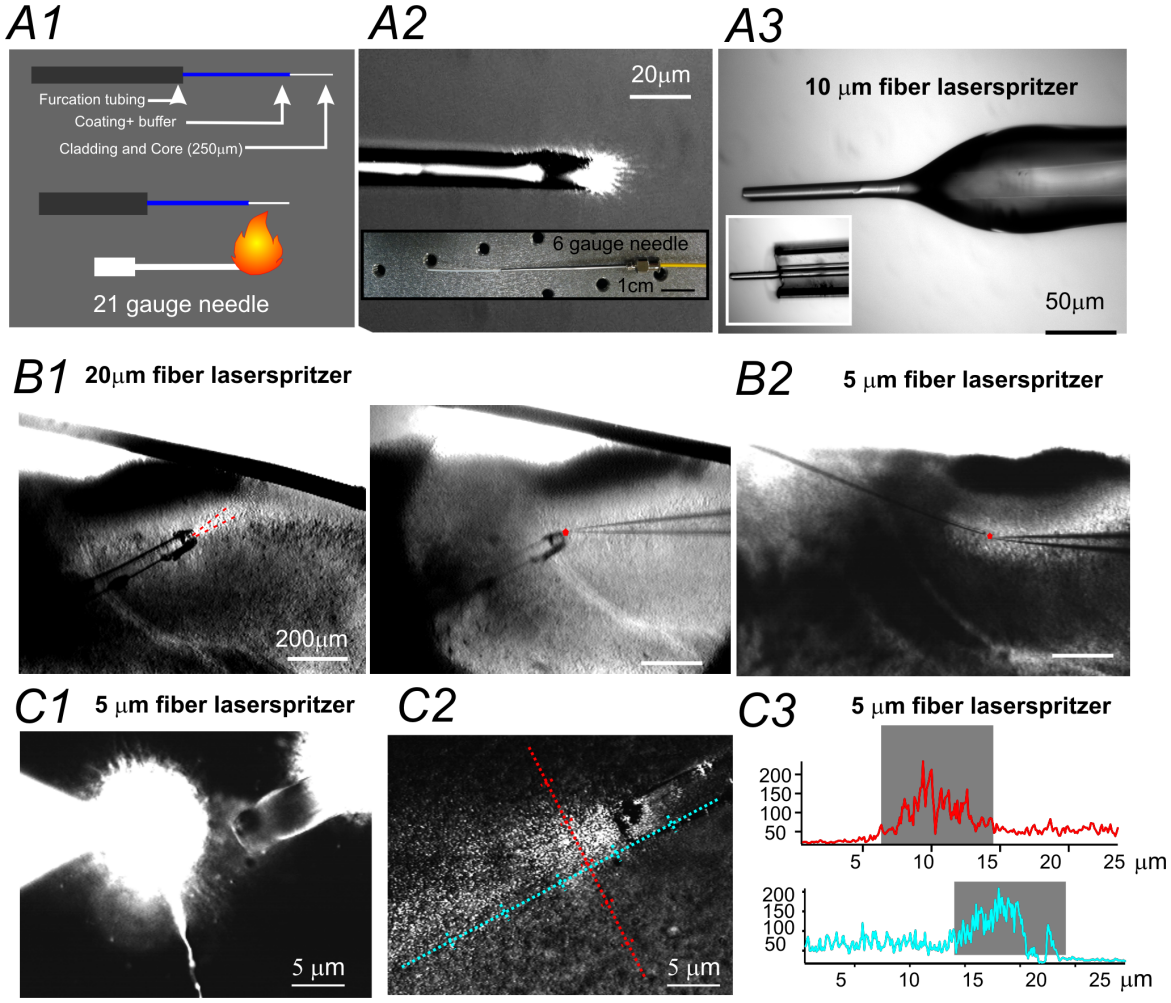
Fabrication and characterization of laserspritzer fiber probe. (**A**). The laserspritzer was created from a multi-mode fiber optic patch cable with an SMA ending. A1–3: Microscopic images of a fiber optic patch cable at different stages of processing. The patch cable was first trimmed and stripped to expose the cladding and core (250 µm). The exposed fiber was heated by a gas burner and gently pulled (A1). The size of illumination of the fiber tip was measured with a microscope (A2) prior to being mounted with a holder, which was made with a 6 gauge needle and a 1cc syringe (A2). The tip of the laserspritzer can be fire polished (A3). (**B**). Photomicrograph of two different laserspritzers placed on two piriform cortex brain slices, respectively. B1: A 20 µm diameter laserspritzer and its laser illumination path indicated by two red dashed lines. B2: A 5 µm diameter laserspritzer placed next to a neuron with a recording pipette (red circle). (**C**) C1: Photomicrograph of a 5 µm laserspritzer placed next to the soma of a neuron that is intracellularly loaded with Alexa594. C2: Laserspritzer illumination by a 470nM laser at a low intensity of 0.04 mW/mm^2^. C3: Line profile of laser intensity crosses the center of the laser beam measured at the same spot; either perpendicular to (red) or parallel to (blue) the laser beam. The area of the laser spot is 10 by 5 µm^2^.

The laserspritzer was made from a multi-mode fiber optic patch cable (e.g. Catalogue Number. M38L02, Ø200 µm, Thorlabs) with appropriate mating ends (e.g. SMA) to the light source (laser or LED, e.g. M490F1, Thorlabs). Step 1. We trimmed and stripped the fiber optic patch cable to expose the cladding and core (250 µm) by about 2–4 cm (Fig. 1A1). Step 2. We heated the fiber core with a homemade gas burner (made with a 21 gauge needle) until it became pliable, then gently pulled the fiber with a pair of micro-Adson forceps (Fine science tools). Step 3. We examined the pulled fiber tip under a light microscope (10X objective) and measured the tip diameter using Zeiss Axiovision (Rev 4.6) software. The sizes of the tip diameters typically ranged between 1 to 30 µm. Fibers with a desirable tip sizes were further tested to examine the light scatter (Fig. 1A2). With some practice, we were able to reliably pull fibers with tip diameters ranging from 5–10 µm. Step 4. We then used a ruby fiber scribe (Thorlabs, S90R) to trim the edge under a dissection microscope. Step 5. If laser emission from the fiber tip was favorable, we carefully inserted the fiber tip into an ‘electrode assembly’ composed of: a syringe, a 6 gauge needle, and a capillary glass pipette tip (Fig. 1A2). Optional Step. The tip of the laserspritzer was polished with a homemade gas burner (Fig. 1A3). Step 6. The finished laserspritzer was mounted onto a micromanipulator (MP285, Sutter Instruments) on a slice electrophysiology rig for further testing. The light scattering of the laserspritzer within a brain slice was tested using a low light CCD camera (Hamamatsu, Model No. C5403, Japan).

Depending on the type of application (stimulation of a large nerve track like a thalamocortical projection or a single axon from a single neuron), laserspritzers with different tip sizes were used (e.g. Fig. 1B1 v. B2). A laserspritzer with desired spot excitation (∼<10 µm spot size with 0.02–0.04 mW/mm^2^ laser power) was collected for further experimentation. Prior to electrophysiology experimentation, we recommend that laser scattering properties be measured in testing brain slices. Fig. 1C shows an example of a testing experiment in a brain slice. The light scatter via the laserspritzer was first imaged with a CCD camera (Fig. 1C2). Then, the laser excitation profile was analyzed using common image analysis software (e.g. image J or Igor Pro). The spot size was defined by using line profiles of the laser beam (Fig. 1C3). A desirable laserspritzer should have an excitation spot of 10–100 µm^2^, at physiologically relevant laser intensities (Fig. 1C3). An advantage of the laserspritzer is that once it is calibrated, it can be repeatedly used in many subsequent experiments (as long as the tip is intact). The laserspritzer was routinely cleaned after each experiment with 70% alcohol and stored on the micromanipulator for subsequent experiments. Prior to every experiment, we recommend that both the laser intensity and light scattering pattern near the tip of the laserspritzer be tested.

## Results and Discussion

### Validation of functional spatial properties in brain slices

**Figure 2:**
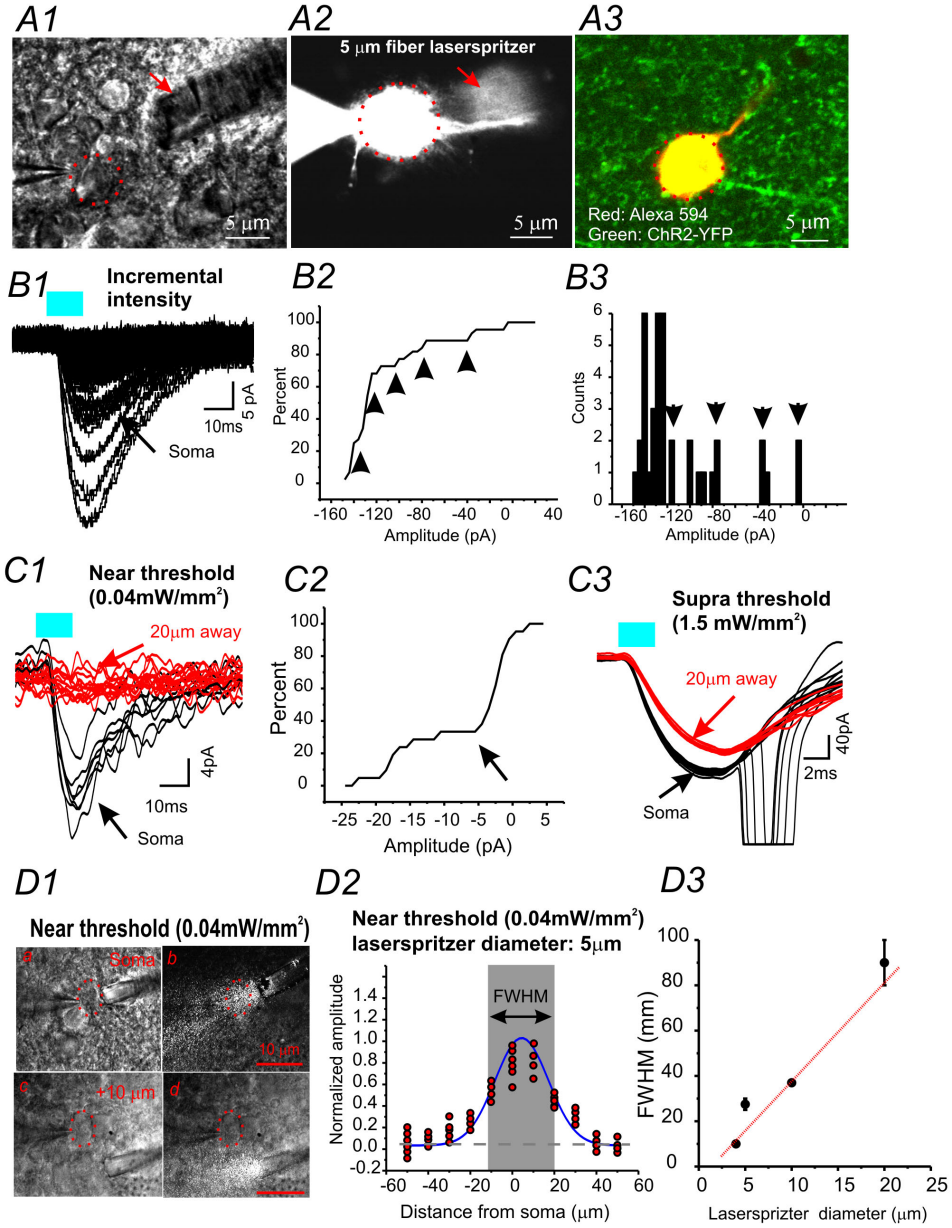
Characterization of the spatial resolution of the laserspritzer using ChR2-expressing interneurons in brain slices. (**A**) High magnification photomicrograph of the D.I.C. (A1) and fluorescent (A2) images showing that the whole-cell recording was made from a ChR2 expressing interneuron (A3), and that a laserspritzer electrode was placed very close to the soma of the recorded cell (A1 & A2). (**B**) Laserspritzer (soma location) induced ChR2 current in a ChR2 positive interneuron. The laser intensities were incrementally increased to induce larger currents. The amplitude of ChR2 currents increased incrementally (arrowheads in B2 & B3). B2 & B3: cumulative probability plot (B2) and histogram plot (B3) of ChR2 current amplitudes. Arrowheads in B2 & B3 indicate incremental increases in ChR2 current amplitudes. (**C**) Laser (490nm, 5 ms) induced by near threshold (C1) and supra-threshold currents (C3) in a ChR2-positive layer I interneuron. The currents were induced by placing the laserspritzer at the soma (black traces) or 20 µm away from the soma (red traces), respectively. C2: Cumulative probability plot of ChR2 current amplitudes. Arrow in C2 indicates failure to induce ChR2 currents when laserspritzer was 20 µm away. (**D**) D1: Photomicrograph of a whole-cell recording in a brain slice showing that a 5 µm laserspritzer electrode is placed near the soma (a & b) or 10 µm (c & d) away from the soma (dotted circle) with (right) or without (left) laser illumination. D2: The laser induced ChR2-mediated current (e.g. C1) is plotted against the location of the laserspritzer with reference to the cell body. FWHM (Full width at half maximum) at this laser intensity (0.04 mW/mm^2^) is 30 µm (n = 6). D3: FWHM-tip diameter plots, dotted line: linear fitting with r^2^ = 0.85.

The spatial resolution of the laserspritzer was examined by making whole-cell recordings from ChR2-expressing cells in brain slices from VGAT-ChR2 mice [5]. Animal work has been approved by the IACUC of the University of Wyoming. We chose cortical layer I ChR2-expressing neuroglia-form interneurons for these recordings, due to the compact cell size and limited dendritic arbors of these cells (Fig. 2A). After identification of the interneuron, whole-cell recordings were made from the identified cell. First, we placed the 5 µm laserspritzer next to the soma of ChR2- expressing interneurons (Fig. 2A1). Under voltage-clamp mode, we gradually increased the laser intensity until a small ChR2 current was induced (Fig. 2B1). Next, we repeated ChR2 current recordings under gradually increasing levels of laser intensity. Our results demonstrate that ChR2 currents increased incrementally with each stepwise increase in laser intensity (Fig. 2B2–3, arrowheads). Next, we used a near threshold laser intensity, repeating the experiments at 0.5Hz for 1 minute (Fig. 2C1). Then, we slowly moved the laserspritzer away from the soma in 10 µm steps, perpendicular to the direction of the laser beam. The procedure was repeated until the laser failed to induce ChR2-mediated inward currents (Fig. 2C1 &2, Fig. 2D1). We then repeated the same experiments using a supra-threshold laser intensity that induced action potentials (Fig. 2C3). Based on the results from both experiments, we determined that the resolution of our laserspritzer (5 µm tip) was 20 to 40 µm (n = 7 cells), depending on laser intensity. The spatial relationship was further examined using FWHM (Full width at half maximum) at near threshold laser intensity; producing a resolution of 30 µm (Fig. 2D). When the laserspritzer was placed at 20±4 µm away from the recorded neurons, less than 10±2% of ChR2 currents were induced at the soma by the near threshold laser. Thus, we concluded that the spatial resolution of the laserspritzer is similar or better than that of the sCRACM [∼40 µm, [12], [13]]. The relationship between resolution and laserspritzer tip size is shown in Fig. 2D3.

A potential shortfall associated with the laserspritzer is that it does not specifically limit the light scattering within the tissue unlike the two photon stimulation method. We typically minimize light scattering with the laserspritzer by decreasing its tip size (Fig. 2D3) and utilizing near threshold intensities (Fig. 2C). In addition, in order to achieve optimal spatial resolutions, the light path of the laserspritzer should be positioned perpendicular to the dendritic arbor (e.g. Fig. 4).

### Application 1. Selective activation of monosynaptic perisomatic inhibitions

Optogenetic methods have been widely used to evoke synaptic releases. Expression of ChR2 within specific presynaptic neurons are achieved by means of virus delivery [23], [25], [26] or genetic targeting [5]. Researchers can selectively activate ChR2 expressing cells with light and study light induced synaptic releases in postsynaptic cells. Various collimated LED illuminations delivered via microscope objectives were widely used as a means to activate ChR2 expressed in brain slices [24]. With this approach, the entire field under a microscope objective would be illuminated. Thus, illumination size is determined by the magnification and optical properties of objective lenses. The exposed area ranges from a few hundred square micrometers (e.g. 63X water immersion lens) to a few square millimeters (e.g. 5X objective). Therefore, a large area of the brain slice is repeatedly stimulated, which will induce desensitization of ChR2. Desensitization is a profuse ramification of repeated light exposure that is a main problem associated with whole-field illuminations.

### Collimated LED illuminations vs. laserspritzer

**Figure 3:**
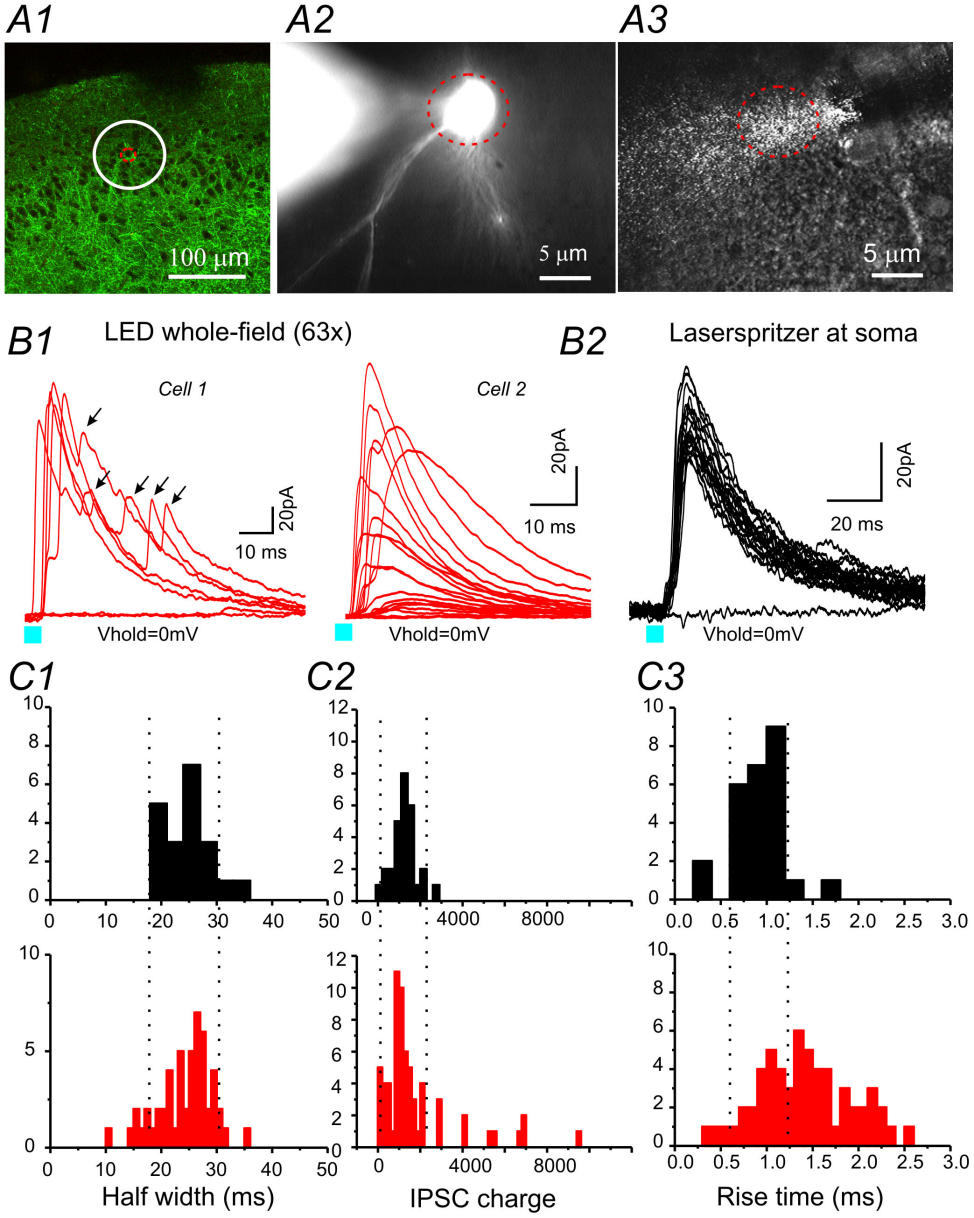
Comparison of GABAergic IPSCs induced by either collimated LED whole-field illumination or laserspritzer in PC slices. (**A**) Laserspritzer stimulation in brain slices. A1: Photomicrograph of VGAT-ChR2 expression within the aPC with the location of the recorded cell (red circle) and the size of the collimated LED whole-field illumination under a 63x water immersion objective (white circle). A2 and A3: High magnification photomicrograph of an Alexa594 filled pyramidal neuron (A2) and the location of laser illumination from a laserspritzer with a 5 µm fiber tip (A3). (**B**) Current traces of ChR2-IPSCs evoked by collimated LED whole-field illumination (B1) vs. a laserspritzer placed very close to the soma of the recorded cell (B2). (**C**) Comparison of the properties of IPSCs induced by collimated LED whole-field illumination (bottom red) vs. laserspritzer (top black). Dotted lines indicate distribution associated with the laserspritzer.

We sought to compare GABAergic inhibitory synaptic currents induced by the laserspritzer to collimated LED whole-field illuminations. Mice used in this study were vesicular γ-aminobutyric acid transporter-channelrhodopsin2 (H134R)-enhanced yellow fluorescence protein mice (hereafter called VGAT-ChR2). In this line of mice, the ChR2-YFP fusion protein was selectively expressed under the control of a VGAT promoter [5]. Mice aged postnatal week 6–8 were used. Whole-cell recordings were made from layer II non-GABAergic cells within the piriform cortex (PC) slice. For GABAergic synaptic currents induced by ChR2 activation, synaptic responses were recorded at a holding potential of 0 mV with a cesium based pipette solution, and were verified pharmacologically via sensitivity to picrotoxin (100 µM, TOCRIS bioscience). Single light pulses (5 ms) were delivered every 20 seconds to prevent the desensitization of ChR2. First, we recorded ChR2-IPSCs mediated by collimated LED whole-field illumination via a 63x water immersion objective. As shown in Fig. 3B1, blue LED light (5 ms pulse) induces robust ChR2-IPSCs in recorded neurons. Next, we used the laserspritzer placed right against the soma of the recorded neuron (Fig. 3A3), and recorded ChR2-IPSCs induced by a 5 ms laser pulse. As shown in Fig. 3B2, the laser pulse reliably induced ChR2–IPSCs in the same neuron. Compared with collimated LED illumination induced ChR2-IPSCs, the properties of the laserspritzer-induced IPSCs appeared more homogenous by half-width, rise time, and area (Fig. 3C top vs. bottom panels). This suggests that the IPSCs were mediated by a homogenous population of synaptic terminals, likely to be perisomatic terminals mediated by basket cells. In contrast, ChR2- IPSCs induced by collimated LED whole-field illuminations appeared heterogeneous, with onsets, latencies, and rise and decay-time appearing more variable (Fig. 3B & C). In several cells, the kinetics of IPSCs within the same cell were more distinct (Fig. 3B1, recordings from cell 2 on the right), suggesting that synapses from different types of interneurons were activated. In addition, multi-peak IPSCs were induced (e.g. Fig. 3B1 black arrows), presumably due to the simultaneous activation of both the soma and terminal of interneurons. In comparison, it is clear that the laserspritzer provides precise activation of monosynaptic perisomatic inhibitory inputs onto recorded neurons. In addition, the laserspritzer possesses another benefit over collimated LED whole-field illumination. In the former method, the activation of ChR2 containing terminals is limited to the light path (around 10–15 µm diameter, e.g. Fig. 1C3). This reduces the unwanted ChR2 desensitization in surrounding areas. This feature is very useful to studies in which a limited number of slices can be produced within the region of interest. Using the laserspritzer, a number of cells separated by 30um can be recorded and stimulated repeatedly from the same slice, without causing desensitization of ChR2. This will reduce the number of animals used and improve sampling efficiency. The introduction of the laserspritzer will require an additional micromanipulator, which may be a slight downfall. It may compete for limited space under the objective that may not be desirable in dual/multiple patch recording experiments. The utility of the laserspritzer in activation of perisomatic GABAergic inputs formed on the soma is demonstrated here. In other recent studies, we used this approach to selectively activate axo-axonic GABAergic synapses (AAS) formed on the axon initial segment (AIS).

**Figure 4:**
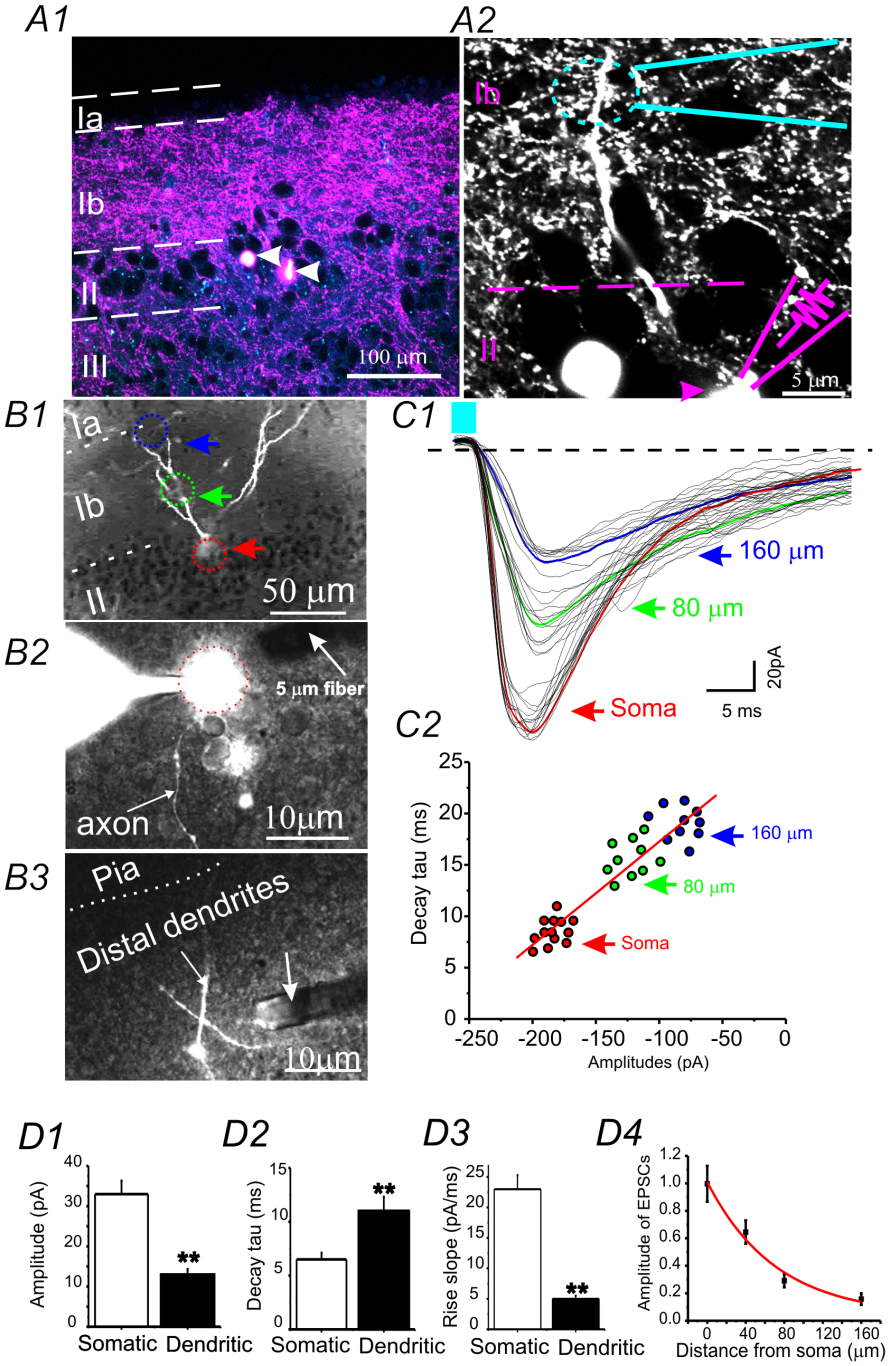
Somatically vs. distal dendritically induced intracortical long-range glutamatergic inputs in an aPC neuron. (**A**) Photomicrograph within the caudal aPC showing the labeling of the rostral to caudal aPC long-range excitatory projections in lower magnification (A1) and higher magnification (A2). Note the location of the long-range input-formed synapses on the distal dendrites of an Alexa594 filled neuron (A2) and the location of the laserspritzer near distal dendrites. (**B**) Photomicrograph showing whole-cell somatic recordings made from the soma of a layer II aPC neuron. B1: A typical semilunar cell with cell body located at aPC layer IIa (dotted red circle). Dotted green and blue circuits and arrows indicate the location of dendritic laser stimulation. B2: Photo showing the soma and axon of a neuron intracellularly loaded with Alexa594 via a patch pipette. The 5 µm laserspritzer (white arrow) is placed near the soma. B3: The 5 µm laserspritzer (white arrow) is placed at distal dendrites (∼150 µm away from soma). (**C**) Dendritic vs. somatically induced monosynaptic EPSCs. C1: Laser induced monosynaptic EPSCs recorded by somatic whole-cell recordings (V_hold_ = −70 mV) in presence of TTX(1 µM) and 4-AP (1 mM). C1: The EPSCs were induced by placing the laserspritzer at somatic (red arrow), or dendritic (80 µm and 160 µm away) locations respectively. C2: Decay time constants of EPSCs were plotted against the amplitudes. Solid red line: line regression of the scatter plot. (**D1**) Comparison of the properties of the dendritic (black bars, 160 µm away from soma) vs. somatic EPSCs (open bars) recorded in 6 aPC neurons. **: p<0.01.

### Application 2. Selective activation of long-range intracortical excitatory synaptic inputs onto distinct dendritic compartments

A potential issue with collimated LED whole-field illumination is that all monosynaptic inputs formed on different cellular compartments are stimulated simultaneously. Thus, the recorded evoked whole-cell ChR2-EPSCs result from the temporal and spatial summation of multiple EPSCs arriving at different subcellular domains (dendrites and soma). This problem is more prominent in cells with large dendritic arbors, such as neocortical pyramidal neurons. To test that our laserspritzer approach can help overcome this issue, we studied long-range intracortical projections formed on different dendritic sites. We chose the olfactory cortex slice because intracortical glutamatergic inputs are formed on specific subcellular domains on distal dendrites in layer IB [30], which is distal from the soma (Fig. 4A2). We injected ChR2-containing AAV viruses (AAV_ChR2_mVenus) in the rostral portion of the anterior PC (aPC) in normal CD-1 mice, which resulted in robust labeling of intracortical long-range glutamatergic axons innervating multiple olfactory brain regions. We took whole-cell recordings in layer II semilunar cells located in the caudal portion of the aPC. This is where robust ChR2 expression can be seen in layer Ib, the known association fiberarea of the aPC (Fig. 4A). Alexa594 (0.2 mg/ml; Invitrogen, A10438) was loaded into the recorded neurons via patch-pipette. After 5 minutes of Alexa594 loading, the entire dendritic and axonal arbors of the recorded cell were readily visible (Fig. 4A2 &B1). Next, we placed the laserspritzer in the soma and different distal dendritic locations (40 µm increments) away from soma (e.g. Fig. 4B &C, respectively), and recorded laser induced EPSCs. TTX (1 µM), 4-AP (100 µM) and picrotoxin (100 µM) was present in the perfusate to allow activation of only monosynaptic glutamatergic inputs [13], [23].

As shown in Fig. 4C1, somatically induced aPC-EPSCs had fixed latencies, fixed onsets, and fast-rise and decay time. In contrast, the distal dendritically induced aPC-EPSCs had a much slower rise slope, decay time, amplitudes, and delayed onsets (Fig. 4C2 &D, n = 6, p<0.01). Comparing stimulation locations with the properties of EPSCs indicate a close linear relationship between amplitude and decay time recorded from different dendritic sites (Fig. 4C2). The EPSC amplitude and dendrite relationship results in a perfect single exponential decay (Ch2 = 0.007, R2 = 0.98, n = 6 cells, Fig. 4D4).

Assuming the distribution of synapses is uniform along all distal dendritic arbors in the aPc (which appears to be true, e.g. Fig. 4A2), the differences between the properties of EPSCs induced dendritically vs. somatically are consistent with idea that axial resistance (Ri) within dendrites can cause a voltage drop between the site of distal inputs and the soma, and that the temporary storage of synaptic charge in membrane capacitance (Cm) slows the kinetics of the recorded EPSCs [31]. In several studies where cellular properties of long-range inputs were studied using sCRACM (subcellular channel rhodopsin assisted circuit mapping) approaches, long-range S1 inputs were found to be distributed across entire dendritic arbors of pyramidal neurons in M1. They were distributed in such a way that the single cell inputs map reflects the dendritic morphology of the pyramidal cell [12], [13], [26]. Thus, the degree of current attenuation induced 150 µm away from soma of aPC neurons was similar to those induced ∼300 µm away from soma in neocortical pyramidal neurons of layer 5 and 6 [32]. This is presumably due to much smaller dendritic diameters in these dendrites and the lack of common primary apical dendrites (Fig. 4B1). Nonetheless, the distinct properties of dendritically vs. somatically induced EPSCs suggest that the laserspritzer achieved synaptic activation with subcellular resolutions. Using this approach, we originated an instance where intracortical association fibers on specific aPC neurons can be selectively activated without the need of pharmacological treatments [30], which have been the standard practice in the field to date. The utility of the laserspritzer in activation of glutamatergic inputs formed on highly confined dendritic compartments is therefore demonstrated here.

## Conclusions

Described here is a fiber optic based simple method of optogenetic stimulation: the laserspritzer approach. This is a convenient and low cost approach that can achieve high spatial resolutions of stimulation and can be easily implemented with an existing slice electrophysiology setup and repeatedly used upon initial validation. Our data, composed of direct ChR2 mediated-current recordings, demonstrates that spatial resolutions created by the laserspritzer lie in the 30 µm range. Using olfactory cortical slices, our data demonstrates that the laserspritzer approach conveys significant advantages over the widely used collimated LED illuminations for both spatial resolutions, and also reduces ChR2 desensitization in brain slices. We provide examples to apply this approach in studying monosynaptic perisomatic GABAergic basket synapses or long-range intracortical glutamatergic inputs formed on different dendritic and somatic domains within the olfactory cortical slices *in vitro*.
